# Development and feasibility study of a Culturally Tailored Asthma intervention using a mixed-method approach at the primary school level in Malaysia: Challenged by the COVID-19 pandemic

**DOI:** 10.51866/oa.675

**Published:** 2025-01-27

**Authors:** Siti Nurkamilla Ramdzan, Ee Ming Khoo, Steve Cunningham, Jayakayatri Jeevajothi Nathan, Nursyuhada Sukri, Hilary Pinnock

**Affiliations:** 1 MBBS, MFamMed, PhD, Department of Primary Care Medicine, Faculty of Medicine, Universiti Malaya, Malaysia. Email: sitinurkamilla@um.edu.my; 2 MBBS, FBSCH, MRCGP, MD, Department of Primary Care Medicine, Faculty of Medicine, Universiti Malaya, Malaysia.; 3 MBChB, MCRP, PhD, NIHR Global Health Research Unit of Respiratory Health (RESPIRE), Usher Institute, The University of Edinburgh, United Kingdom.; 4 BSc, MSc, PhD, Department of Primary Care Medicine, Faculty of Medicine, Universiti Malaya, Malaysia.; 5 MMedSc, Department of Primary Care Medicine, Faculty of Medicine, Universiti Malaya, Malaysia.; 6 MBChB, MRCGP, MD, NIHR Global Health Research Unit of Respiratory Health (RESPIRE), Usher Institute, The University of Edinburgh, United Kingdom.

**Keywords:** School health services, Asthma, Feasibility studies, Child

## Abstract

**Introduction:**

The World Health Organization recommends incorporating asthma programmes into national school health services, although this recommendation is rarely implemented.

**Methods:**

In Malaysia, we developed a multi-level primary school asthma programme incorporating educational sessions for children with asthma and their parents, raising awareness within the whole school community and training school staff to provide first-aid asthma management. The programme was adapted for delivery during the COVID-19 pandemic, and a mixed-method feasibility study was conducted in October 2020.

**Results:**

We identified 34 children with asthma, who comprised 3.7% of the school population. Only 14/34 (41.2%) children with asthma and 4/14 (28.5%) of their parents attended the remote sessions. The in-person session for school staff was attended by 55/62 (88.7%), among whom 86.0% rated the session as good/excellent.

**Conclusion:**

The school-based intervention was feasible and received good feedback, despite the COVID-19 pandemic forcing remote delivery. Stakeholder engagement is essential in the development and feasibility of a school-based asthma programme.

## Introduction

For more than 25 years, the World Health Organization (WHO) has promoted child, adolescent and community health via school-based programmes.^1^ Globally, over 90% of children attend primary school with health-promoting school initiatives offering an ‘easy entry point’ for children to have access to healthcare.^1^ Schools are important resources for instilling healthy behaviours at a young age and, by extension, influencing families.^2^ The WHO initiative has successfully implemented communicable disease health-promotion programmes (e.g. deworming, vaccination, water, sanitation and hygiene) in low- and middle-income countries.^2^ Non-communicable diseases are a challenge in all economies, but health programmes have been more successful in high-income countries. In June 2021, the WHO and United Nations Educational, Scientific and Cultural Organization (UNESCO) published guidelines on ‘Making Every School a Health-Promoting School’, which recommended communicable and non-communicable disease programmes in schools. As part of health-promoting school initiatives, supporting care and first-aid training for asthma are recommended as essential school health services in all countries.^2^ However, supporting asthma care and training of school staff are not included in the Malaysian national school health services guideline.^3^ Our previous studies found that children with asthma received poor support for self-management at school, which could delay treatment during emergencies.^4,5^ Misperceptions that may stigmatise these children are common, highlighting the need for interventions to improve their well-being in Malaysia.^4,5^

Therefore, we developed a Culturally Tailored school-based intervention for Asthma in Malaysia (CuT-AsthMa) for primary schoolchildren and assessed its feasibility.

## Methods

We used the UK Medical Research Council complex intervention framework^6^ to develop the CuT-AsthMa programme and engaged with multiple stakeholders including children with asthma and their parents, school staff, healthcare professionals and policymakers throughout the different phases of the study.

### CuT-AsthMa programme

The CuT-AsthMa programme was developed based on a systematic review^7^ and qualitative exploration of school-based asthma programmes among children with asthma, their parents and other important stakeholders.^4,5,8^ From these, we learnt that it is crucial to involve children’s social networks and address common misconceptions in the community. Together, we co-designed a simple, practical and fun programme. Table 1 describes the CuT-AsthMa programme using the Template for Intervention Description and Replication checklist.^9^

**Table 1:** Description of the CuT-AsthMa programme using the TIDieR checklist.

TIDieR item	Description
Title	CULTURALLY TAILORED SCHOOL-BASED INTERVENTION FOR ASTHMA IN MALAYSIA (CuT-AsthMa)
Why	The intervention aimed to provide asthma education to the school community.
What	There were three key elements of the programme: 1. Frequent brief recommendations and reminders about asthma were provided to raise awareness within the school community (targeted at children with asthma, school staff and all schoolchildren). The messages focused on asthma symptoms and addressed common misperceptions about asthma (e.g. Asthma is contagious’ and ‘Children with asthma cannot play sports’). We used various modes of communication, such as posters, pamphlets and school media systems including the public awareness system. Electronic materials were sent to parents via school communication channels, such as the school/class WhatsApp group. The purpose of these brief recommendations and reminders was to promote participation in the programme and create an asthma-friendly school environment. 2. A generic school asthma action plan targeted at school staff was implemented to help them manage asthma emergencies in school (Supplementary Material 1). We adapted the plan from the personalised asthma action plan recommended by Malaysian and other guidelines for self-managing asthma symptoms and attacks.^4^ Our prior qualitative studies suggested that it is unrealistic to expect each child to have their own personalised asthma action plan available in school.^5^ Parents rarely communicated individualised plans they had been given at home (although if it were available, it would take precedence over a generic school plan). Providing children with individual plans needed the assistance of a designated school nurse, which was not available in government schools in Malaysia.^4,10^ A generic school asthma action plan was a pragmatic approach that summarised general actions needed by school staff to act appropriately if an asthma event occurred in school. 3. Educational videos aided in delivering and disseminating several aspects of the intervention, creating awareness and reducing stigma among all members of the school community.^11^ More specifically, they supported inhaler technique training sessions for children with asthma and school staff.
Who provided	The research team consisted of a primary care specialist trained in childhood asthma.
How	The programme was delivered as part of the health awareness programme in the school. Educational sessions were provided for children with asthma and their parents, raising awareness within the whole school community and training school staff to provide first-aid asthma management in line with a school plan as part of a continuous professional development training programme. Interactive group sessions were conducted to provide the key messages of the programme, involving hands-on training in small groups.
Where	The programme was conducted in school (online delivery is considered to conform to current COVID-19 restrictions).
When and how much	We delivered two sessions lasting about 1 hour for children with asthma and their parents. For school staff, the programme consisted of a training session lasting for 2 hours. • To ensure good participation, we planned the delivery of the programme with the school administrators (e.g. after school).
Tailoring	We worked in partnership with stakeholders (e.g. teachers, children with asthma and their parents and healthcare professionals) to refine the materials and implementation strategy of the programme. We adapted the programme to conform to COVID-19 restrictions.
Fidelity assessment	During the hands-on training in small groups, the facilitator assessed whether participants could understand the key messages and demonstrate first-aid asthma care. A notetaker was present to observe and note if participants attended the whole session.

CuT-AsthMa, Culturally Tailored school-based intervention for Asthma in Malaysia; COVID-19, Coronovirus disease 2019; TIDieR, Template for Intervention Description and Replication checklist

Informed by our prior work,^4,5,7,8^ the content aligned with the Malaysian guideline recommendations for asthma management and other global guidelines.^10,12^ Materials such as educational posters and instructional videos were designed through team discussions and meetings with local experts, children and their parents and teachers to ensure face validity. Children’s involvement was particularly important when designing the programme. They suggested simple, lun materials as well as the incorporation of children’s images and voices into the materials. Some children volunteered to use their images and voices. Cartoon images were replaced with children’s images on the posters, and children acted and narrated in the videos.

**Figure 1:**
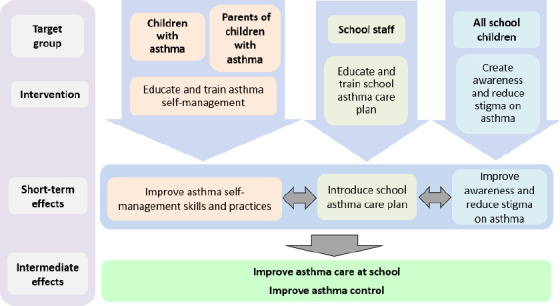
Logic model of the CuT-AsthMa programme guided by the socio-ecological theory.^13^

**Figure 1** shows the logic model of the programme. In three parallel streams, the CuT-AsthMa programme targeted children with asthma and their parents, school staff and all schoolchildren with an education and skills programme designed to improve children’s self-management, school’s management of asthma emergencies and general awareness within the school community.

**Figure 2:**
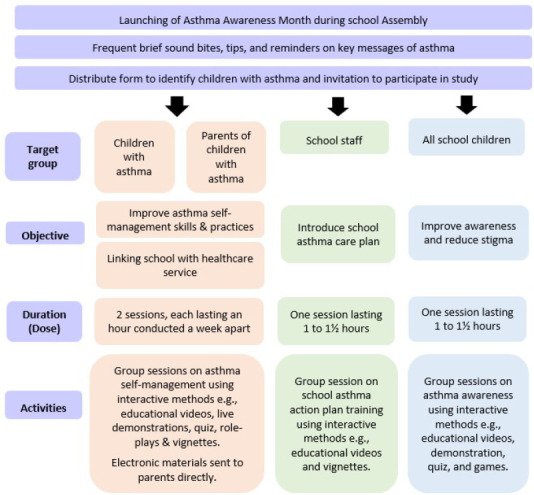
Core components, objectives and activities of the CuT-AsthMa programme.

The programme was developed in Malay and was designed to be delivered as an ‘asthma awareness month initiative’ incorporated into the schools annual health programme. **Figure 2** illustrates the core components, objectives and activities of the programme.

### Identification and recruitment of participants

All intervention activities were intended to be conducted in the school, and we collaborated with the school administrators to identify participants. We provided a participant information sheet iPIS) to all potential participants and stressed that participation was voluntary and that participants were free to withdraw from the study at any time without giving a reason. The PIS also included the researchers’ contact details, allowing participants to reach out with any questions or concerns they might have.

**Children with asthma and their parents**

To identify children with asthma, we sent a screening letter to all parents via the schoolchildren. Parents self-reported if their child had been diagnosed with asthma and was on medication (e.g. prescribed with inhaler/oral medication or used a nebuliser) or had asthma symptoms in the past 1 year. We contacted parents of children who met the eligibility criteria and invited them and their children to join the sessions.

**School staff**

We sent an invitation letter to all teachers to inform them about the session, which was organised as part of the continuous professional development training programme. We excluded school staff without a role in caring for children (e.g. security staff and cleaners).

**All schoolchildren**

Together with the screening letter, we informed all children if the school about the programme, providing an opportunity for parents to opt out if they did not wish their child to participate in the activity. With the assistance of the school administration, the letters were also shared in the WhatsApp group of each class.

### Procedure and adaptation for the COVID-19 pandemic

The feasibility study of the CuT-AsthMa programme was conducted at a school located in a suburban area near Kuala Lumpur in October 2020. We chose the school based on the number of students with asthma, the primary language used in the school and our previous collaboration with the school. The study was led by a researcher, SNR, who was a family medicine specialist trained in the management of childhood asthma. She and her team delivered the intervention to school staff in a face-to-face session. Unfortunately, in the midst of delivering the month-long programme, cases of COVID-19 infection increased, and all schools were closed until the end of the academic year. We then adapted the face-to-face sessions for children with asthma and their parents to online delivery (via Skype). SNR conducted all online sessions assisted by the team members. The posters and short reminders were delivered for 2 weeks instead of a month, and the educational session for all schoolchildren was cancelled. We worked in partnership with the school administration and teachers to make these necessary adaptations.

### Data collection and analysis

The feasibility study was conducted to assess whether the programme could be implemented in a school setting. To understand the enablers and challenges of delivering the programme, we used a quantitative approach to measure participation rates and gather feedback from adult participants. A qualitative approach was also employed to obtain feedback from children with asthma.

**Quantitative data**

We collected quantitative data filled in by participants or children’s parents (Supplementary Material 2). The quantitative data were analysed descriptively to illustrate the feasibility of conducting the study. An inferential statistical analysis was not performed, as this study was underpowered to show a significant difference.

**Qualitative data**

We undertook focus group discussions to obtain feedback from children with asthma. The topic guides were developed by the research team based on the socio-ecological theory^13^ and the programme’s content and delivery. The programme was pilot-tested with five children aged 5–11 years, and a question was added to assess preferences for the delivery mode of the session, but no other changes were made. The focus group discussions were conducted and analysed in Malay. The quotes used in the report and manuscript were translated into Malay by SNR, who was proficient in Malay and English, and checked by another bilingual researcher, NS, to ensure accuracy. SNR was an academic family medicine specialist in Malaysia who had over 15 years of working experience. She conducted all the qualitative interviews.

A thematic approach was used for the analysis of the qualitative data, guided by the research objectives and conducted with reference to key concepts related to implementing interventions. SNR read the transcripts multiple times for data familiarisation, created codes and merged similar codes to develop themes. Memos, field notes and discussions with a multicultural team of primary care physicians and a paediatric pulmonologist from the UK and Malaysia were utilised to ensure a balanced interpretation. The research team aided in the data interpretation. The quotes that best captured the essence of the themes were extracted for presentation in the results section.

## Results

The school sent the screening letters to 917 students, and 683 (74.5%) were returned. Only 0.2% (2/917) of the parents did not want to reveal their child’s asthma status, and 2.3% (21/917) of the screening letters were returned blank. We identified 5.2% (48/917) children with asthma, and only 3.7% (34/917) of them had received treatment from a clinic or hospital in the past year.

### Participation in and feedback on the sessions for the children with asthma and their parents

We invited 34 children with asthma to participate in the online sessions. Nine (27%) parents were uncontactable, and 11 (32%) declined participation of their child due to a lack of internet facilities or inconvenient timing, leaving just 14 (41%) able to attend. Only four parents joined the session with their child. Table 2 shows the characteristics of the 14 children who participated in the sessions. The children’s age ranged from 8 to 12 years, and 64% (9/14) had either partly controlled or uncontrolled asthma. More than a quarter had a history of an asthma attack in the past year needing medical attention, and 64% (9/14) had a history of hospitalisation due to asthma.

**Table 2:** Characteristics of the children with asthma (n=14).

Characteristics	n (%)
Age range (mean±SD)	10.1±1.2 years
Duration of asthma (mean±SD)	5±3.4 years
Lower primary (7–9 years)	5 (35.7)
Upper primary (10–12 years)	9 (64.3)
Male	6 (42.9)
Female	8 (57.1)
Malay	10 (71.4)
Indian	3 (21.4)
Iban	1 (7.1)
Asthma symptom control	
Well controlled	5 (35.7)
Partly controlled	8 (57.1)
Uncontrolled	1 (7.1)
History of an asthma attack	
Past 3 months	3 (21.4)
Past 1 year	4 (28.5)
History of hospitalisation	9 (64.3)
Physical activity (hours per week)	
Low (0–1 hour)	1 (7.1)
Moderate (2–3 hours)	6 (42.9)
High (≥4 hours)	7 (50.0)
Preventer asthma medication	
Inhaled corticosteroid	5 (35.7)
Oral leukotriene	1 (7.1)
None	8 (57.1)
Reliever asthma medication	
Inhaled short-acting beta-2-agonist	10 (71.4)
None	4 (28.6)
Brings a reliever inhaler to school	3 (21.4)
Confident in self-managing asthma independently	7 (50)
Has an appointment date for the next asthma review	7 (50)

We invited all children who attended the sessions to provide their feedback in a group setting, and 36% (5/14) agreed. Overall, the children viewed the sessions as enjoyable and could comprehend the key messages. One child said, *‘Nice because I can learn*. Three described the live demonstration of the inhaler technique as easy to understand, and one child explained, ‘I *used it before’,* as the reason for comprehension. A child liked the ‘what is asthma’ activity, as it was simple. He said, ‘There *are not too many things that we need to know, so it is easier*. The children viewed the sessions as important for other children with asthma to learn more about their illness. To make the sessions more interesting, one child suggested more quizzes, and another suggested having more images, videos and stories. Three children would have preferred face-to-face sessions in school, which they thought would be more fun than online sessions. The other two children preferred online sessions, and one explained, *‘Because when we are at school, sometimes we feel tired. Sometimes we have sports’.*

The four parents who attended viewed the sessions as good, engaging and relevant. No parents had attended an asthma education before the sessions.

All parents understood the content and agreed that Malay was the suitable language for delivering the sessions. All parents agreed that the sessions should be conducted yearly and rolled out to other schools. One parent had a technical issue during one of the sessions and was not able to participate in the whole session.

### Participation in and feedback on the session for the school staff

Eighty-nine percent (55/62) of the teachers attended the face-to-face session conducted at school. Five percent (3/62) left (about 15 minutes before the end of the session) due to other commitments. Seventy-eight percent (43/55) provided feedback on the session. Most (86%) viewed the session for staff as good/excellent, engaging and relevant and understood the content. Only 2% (1/43) viewed the session as poor. Approximately 93% (40/43) of the teachers had not attended an asthma education previously, and 67.4% (29/43) considered that the topic was important and that the session should be conducted annually as a reminder/update. A third (14/43) did not think a yearly update was necessary, and only 2.3% (1/43) suggested that the session should be longer. Additionally, 88% (38/43) of the teachers considered that the school action plan could be successfully implemented.

### Barriers and facilitators of remote delivery

Table 3 summarises the barriers and facilitators of remote delivery of the programme. Our greatest challenge was conducting the remote session for the children and their parents due to limited internet access, technical challenges and limited engagement of children and parents. We found many disadvantages of remote sessions among the children. Many younger children did not own personal electronic devices such as computers, tablets or mobile phones for the sessions, which could be the reason for lesser participation among younger primary schoolchildren. They depended on their parents, and some shared the device with their siblings, who may have had an overlapping online session.

**Table 3:** Challenges and enablers of remote delivery.

Challenges	Enablers
Age range (mean±SD)	10.1±1.2 years
Limited internet access among children	Good partnership with the school
Technical issues	Small number of children
Limited engagement of participants	Participation of parents
Disadvantages	Advantages
Low participation of children from a lower socioeconomic background	Practical approach during the pandemic
Unsuccessful remote assessment for scoring the inhaler technique	Low cost

The platform we used did not allow concurrent break-up sessions, and we could not provide the small group activities as planned. An important part of the face-to-face session was the breakup small group activity (not more than a 1:3 ratio) for the inhaler technique training. Remote assessment for scoring the inhaler technique was unsuccessful because either some children were not able to switch on their video, or the researcher had an obscure view to score the inhaler technique. The impact of training on the inhaler technique was therefore not formally assessed.

It was difficult to engage with the children and their parents via the online sessions. We conducted quizzes, which involved the children answering by showing their hands up; this was a problem, as not all children could switch on their cameras. We hoped that remote sessions would make it easier for parents to participate than face-to-face school-based sessions. However, it was difficult to arrange the sessions at a time that was convenient for both children and parents, because they had different commitments. Thus, the sessions were conducted following the children’s preferences.

### Enablers of the CuT-AsthMa programme

There were a few enablers to delivering the programme. A good partnership with the school was crucial and resulted in a good response rate from the screening letters sent to all the schoolchildren. The school used different communication media to inform the school community about the study (e.g. letters to inform parents via their children, reminders to parents and school staff using posters, the school’s group phone messages and the public address system). Due to the small number of children with asthma, we could provide a spacer for each child and a placebo inhaler for those without an inhaler to practise with at home. The remote sessions involving three or fewer children allowed more interaction, as less background noise interfered with the sessions (the children’s microphones were enabled throughout the session). Smaller groups also enabled more personalised training on the inhaler technique. The presence of parents during the sessions encouraged the children to speak out more.

The school’s assistance and cooperation were the main enablers of the teachers’ session, which was conducted successfully as planned. The physical distancing and wearing of masks could have reduced the interaction between the team and the participants, but the response rate and feedback were good.

## Discussion

We successfully developed the CuT-AsthMa programme, but conducting the feasibility study during the COVID-19 pandemic was challenging. The session for the school staff was conducted as planned and well-received. However, the pandemic forced the adaptation of the programme, and the delivery of the sessions for the children with asthma and their parents was converted from face-to-face to online. Despite poor participation, we received good feedback from the online participants. Stakeholder engagement and good partnership with the school were essential enablers of the programme.

The session for the school staff was successful because it was delivered as part of the continuous professional development training programme. This strategy is commonly used in other school-based asthma studies and attracts good participation.^14^ In line with previous studies, we found that the teachers had no or minimal previous training to manage students with asthma in school.^15,16^ A Cochrane systematic review found that school staff who received asthma training were more adherent to asthma policies and were better prepared to manage students developing asthma symptoms in school.^17^ Currently, we are scaling up the programme in collaboration with policymakers from the education and health sectors to develop national guidelines to enhance the detection of asthma symptoms in children and support their self-management.^18^ This is important because good adherence to a school asthma plan and support for children with asthma could reduce morbidities and mortalities.^19^

The COVID-19 pandemic forced the conversion of the face-to-face sessions for the children with asthma and their parents to online sessions, which were partially successful. An intervention designed as online from the beginning would have optimised the benefits of the digital mode of delivery, but due to limited time and resources, our only practical approach was to adapt the content we had designed for face-to-face delivery. Online delivery enables education to be maintained despite social distancing requirements and is described as having advantages of flexibility, low cost and convenience.^20^ However, we found that conducting the online sessions with the children was challenging, resulted in poor attendance and disadvantaged children from lower economic backgrounds. Reflecting this, only a few online school-based asthma programmes have been developed for primary schoolchildren^21^ because of concerns about poor internet access, short attention spans and parental worries about possible harms related to the long screen time.^22,23^ Remote education is often viewed as an alternative when face-to-face sessions are impossible (as in a pandemic) but may not be a suitable long-term solution for young children.^22,23^ Parents and teachers have raised concerns that online learning could cause social isolation, depriving opportunities for social interaction and participation, and delay or reduce feedback from their teachers.^23^ From this study, we learnt that children and parents have different commitments. Holding separate sessions for each group may improve participation in online sessions. Additionally, children’s participation could be enhanced by conducting the programme at school and providing them access to devices and internet.^24^ Future research is needed to understand how remote asthma sessions can be delivered effectively to children especially those from deprived backgrounds as an alternative or update to face-to-face sessions.

In contrast, a remote school-based programme may be more suitable for adults (e.g. parents, school teachers and healthcare professionals).^25,26^ Online first-aid asthma training for school teachers and nurses is common and provides flexibility for participants to attend the training.^17^ Parental participation in previous remote school asthma programmes varies but usually increases with multiple invitations, which could be a good alternative for parents who could not participate in the face-to-face sessions with their children.^24^

The involvement of children is a core value for health research in this age group, as they can offer unique, invaluable expertise to identify research priorities, enhance the study design and increase recruitment.^27^ Programme materials developed together with children are more appealing and could create peer role models for other children.^28^ Involving children as co-researchers could create children’s future interest in research and improve their personal and community development.

Similar to other findings, a good partnership with the school was a crucial enabler for the delivery of the school-based programme.^29^ Interventions developed without school involvement and imposed on schools would be unlikely to lead to unsuccessful implementation. Outlining programmes’ core components allows flexibility and helps maintain the fidelity of programmes while enabling adaptation to ensure successful implementation in diverse school settings.^30^

## Strengths and limitations

The study provides valuable information on the feasibility of delivering some core components of the programme and providing important insights into the challenges of delivering remote sessions to primary schoolchildren. A good partnership with the school enabled good participation in the teachers’ session and positive feedback.

The school-wide survey identified a significant number of children with asthma. The study relied on parent-reported physician-diagnosed asthma, which may be inaccurate. An objective measurement (e.g. spirometry) could improve accuracy but is costly, time-consuming and not appropriate during the pandemic. We co-designed the 1-hour sessions with the stakeholders to ensure that concentration could be maintained and accommodate the tight school schedule. Longer sessions may be needed for more in-depth training.

Due to school closure during the pandemic, the delivery of the core component ‘creating an asthma-friendly school’ was impossible, compelling remote delivery of the sessions for the children with asthma and their parents. We faced many challenges, including poor participation, as remote delivery discriminated against those with limited technology, potentially increasing inequity in access to care. Younger children were underrepresented in the sessions, possibly for the same reason. Those who attended may not be typical of children with asthma and their families.

The sessions were initially planned to be delivered face-to-face and were adapted for remote delivery when the pandemic hit. We were unable to obtain any clinical outcomes, as the programme was not delivered as initially intended. However, the challenges faced provide valuable experiences and learning for planning future studies. Further refining and re-testing of the programme are needed to inform a future trial.

## Conclusion

Schools can play an important role in assisting children to keep their asthma under control by implementing well-coordinated school asthma programmes. The involvement of children in health research and a good partnership with schools are important enablers of school-based health programmes. These strategies are crucial to achieving the global standards for health-promoting school initiatives, as outlined by the WHO and UNESCO.

## Data sharing statement

The datasets generated and/or analysed during the current study are available from the corresponding author upon reasonable request. Some materials from the CuT-AsthMa programme are available at https://www.ed.ac.uk/usher/respire/news/insights/respire-malaysia-instructional-videos.

**How does this paper make a difference in general practice?**

- The programme developed in this study may benefit children with asthma, as there are no school-based asthma programmes in Malaysia.
- Support for teachers and school administration in asthma management particularly during asthma emergencies is crucial.
- The study emphasises the importance of involving stakeholders particularly children in health interventions.
- The study highlights the challenges in delivering school-based asthma programmes remotely for children.
